# Supplementary material for: Content-rich biological network constructed by mining PubMed abstracts
Source: BMC Bioinformatics. 2004 Oct 8;5:147. doi: 10.1186/1471-2105-5-147 (PMC528731; doi:10.1186/1471-2105-5-147)
Supplement: Additional File 2 — The original results of the above study (non-essential files are deleted to keep the file size under the limit set by BMC bioinformatics). [file 1471-2105-5-147-S2.bz2 › chilibotAdditionalFile2/dip30/400ID8955093E9955/html/LIF_LIFR.html]

 


 **LIF** and **LIFR** 
  
Found 183 abstracts in PubMed, retrieved 30.  
 

 What does Google say? 
 PDF only 
| .edu only 

---

**Interactive relationship** (e.g. stimulation, inhibition, etc)

**Inhibitory relationship**- The neurokine leukemia inhibitory factor  [ **LIF** ]   **LIF**  initiates signaling through heterodimerization of the low affinity  **LIF**  receptor  **LIFR**  and gp130.  Ref: 12425940 Brain Res Mol Brain Res, 2002
- The CDFs leukemia inhibitory factor  [ **LIF** ]  and ciliary neurotrophic factor bind to a  **LIFR**  gp130 receptor complex to activate Jak signal transducers and activators of transcription and Ras mitogen activated protein kinases signaling pathways.  Ref: 12609984 J Biol Chem< MedlineTA>J Biol Chem, 2002
- Leukemia inhibitory factor  [ **LIF** ]   **LIF**  promotes giant cell differentiation in vitro, and  **LIF**  receptor  **LIFR**  deficiency results in loss of giant cell differentiation in vivo.  Ref: 12554639 EMBO J, 2003
**Neutral relationship**- Previous studies suggested that the NH 2 terminal CBM and the Ig like domain of the  **LIFR**  are most important for  **LIF**  binding and activity.  Ref: 12731865 Biochemistry, 2003
- Deletion mutants of the  **LIFR**  revealed that both the NH 2 terminal CBM and the Ig like domain of the  **LIFR**  are involved in  **LIF**  binding, presumably via the  **LIF**  site III epitope.  Ref: 11812136 J Mol Biol, 2002
- ... RILLIFdeltaII binds  **LIF**  and mediates signalling as efficiently as the wild type  **LIFR** .  Ref: 11812136 J Mol Biol, 2002
- In analogy to a recently published model of the IL 6R complex, a model of the active  **LIFR**  complex is suggested which positions the COOH terminal CBM at  **LIF**  site I and the NH 2 terminal CBM and the Ig like domain at site III.  Ref: 11812136 J Mol Biol, 2002
- These data indicate that although  **LIF**  and Hyper CNTF use the same heterodimeric receptor complex of gp130 and  **LIFR** , only Hyper CNTF induces neuronal differentiation.  Ref: 12071967 Eur J Biochem, 2002
- Our results indicate that  **LIF** , CT 1 and OSM share an overlapping binding site located in the Ig like domain of  **LIFR** .  Ref: 12707269 J Biol Chem< MedlineTA>J Biol Chem, 2002
- Oncostatin M can activate two related receptors, one consisting of a low affinity  **LIF**  receptor subunit,  **LIFR**  beta, which forms a heterocomplex with the gp130 signal transducing protein and can recognize both oncostatin M and  **LIF** , and a second heterocomplex consisting of a subunit that specifically recognizes oncostatin M, OSMR beta, and the gp130 protein.  Ref: 12061840 Cytokine, 2002
- The different behavior of  **LIF**  and CT 1, on one side, and of OSM, on the other side, can be related to the different affinity of their site III for  **LIFR** .  Ref: 12707269 J Biol Chem< MedlineTA>J Biol Chem, 2002
- Signal transduction by CNTF requires that it bind first to CNTF R, permitting the recruitment of gp130 and  **LIF**  R  [ **LIFR** ] , forming a tripartite receptor complex.  Ref: 12765290 J Zhejiang Univ Sci< MedlineTA>J Zhejiang Univ Sci, 2002

**Non-interactive relationship** (e.g. studied together, co-existance, homology, etc.)

- While on BAF3 gp130  **LIF**  R  [ **LIFR** ]  cells, Hyper CNTF and  **LIF**  exhibited similar biological responses, the activity of Hyper CNTF on pheochromocytoma cells PC12 cells was quite distinct from that of  **LIF** .  Ref: 12071967 Eur J Biochem, 2002
- gp130 either homodimerizes in response to IL 6 and IL 11 or forms heterodimers with the leukemia inhibitory factor  [ **LIF** ]   **LIF**  receptor  **LIFR**  in response to  **LIF** , oncostatin M OSM, ciliary neurotrophic factor CNTF, cardiotrophin 1 CT 1 or cardiotrophin like cytokine resulting in the onset of cytoplasmic tyrosine phosphorylation cascades.  Ref: 12047380 Eur J Biochem, 2002
- Pinopode formation and expression of  **LIF**  and  **LIFR**  were examined in endometrial samples.  Ref: 12620495 Fertil Steril, 2003
- Leukemia inhibitory factor  [ **LIF** ]  receptor  **LIFR** , epidermal growth factor receptor EGFR, and their respective ligands have been implicated in regulating growth and development of the early pig conceptus.  Ref: 12698007 Cytogenet Genome Res, 2002
- **LIF**  receptors, gp130 and  **LIFR**  alpha were significantly upregulated in cHI P &lt.  Ref: 12799551 AIDS< MedlineTA>AIDS, 2002
- Serum  **LIF**  R  [ **LIFR** ]  concentrations were significantly increased 2 and 4 months after starting treatment with atypical antipsychotics.  Ref: 11950553 Schizophr Res, 2002
- We measured plasma kynurenine and tryptophan and immune markers, such as serum interleukin 6 IL 6, IL 8 and the leukemia inhibitory factor  [ **LIF** ]  receptor  **LIF**  R  [ **LIFR** ]  in healthy, nonpregnant and pregnant women at the end of term and one and three days after delivery.  Ref: 12175700 Life Sci, 2002
- Normal human pituitary tissue expressed  **LIF** , as well as the  **LIF**  receptor  **LIFR**  signaling subunits, gp130 and  **LIFR** .  Ref: 12574225 J Clin Endocrinol Metab, 2003
- CONCLUSION S The simultaneous positive spatial and temporal expression of pinopodes and  **LIF**  and  **LIFR**  proteins in endometrial samples from healthy women suggests that both molecular and structural cell changes are important in the initiation of human blastocyst implantation.  Ref: 12620495 Fertil Steril, 2003
- Molecular modeling of the complex of  **LIF**  with the Ig like domain of  **LIFR**  provides a clue for the superadditivity of the D214A F284A double mutation.  Ref: 12707269 J Biol Chem< MedlineTA>J Biol Chem, 2003
- In addition, the expression of ciliary neurotrophic factor receptor CNTFR and leukemia inhibitory factor  [ **LIF** ]  receptor  **LIFR**  mRNAs, but not of interleukin 6 receptor mRNA, was up regulated after systemic lipopolysaccharide treatment.  Ref: 12127095 Neuroscience, 2002
- Cells that only express gp130 and  **LIF**  R  [ **LIFR** ] , but not CNTF R are refractory to stimulation by CNTF.  Ref: 12765290 J Zhejiang Univ Sci< MedlineTA>J Zhejiang Univ Sci, 2002
- The functional receptor complex of CNTF is composed of the CNTF receptor alpha CNTFR, gp130 and the leukemia inhibitory factor  [ **LIF** ]  receptor  **LIFR** .  Ref: 11943154 FEBS Lett, 2002
- We concluded that the fusion protein Hyper CNTF had superagonistic activity on target cells expressing gp130 and  **LIF**  R  [ **LIFR** ] , but lacking membrane bound CNTF R.  Ref: 12765290 J Zhejiang Univ Sci< MedlineTA>J Zhejiang Univ Sci, 2002
- Human CNTF elicits cellular responses by induction of a receptor complex consisting of the CNTF alpha receptor CNTFR, which is not involved in signal transduction, and the beta receptors gp130 and leukemia inhibitory factor  [ **LIF** ]  receptor  **LIFR** .  Ref: 12643274 J Biol Chem, 2003
- This study was carried out to examine 1 the serum concentrations of interleukin 8 IL 8, IL 10, the soluble CD8 sCD8 and the leukemia inhibitory factor  [ **LIF** ]  receptor  **LIF**  R  [ **LIFR** ]  in nonresponders to treatment with typical neuroleptics as compared with normal volunteers and responders to treatment.  Ref: 11950553 Schizophr Res, 2002
- Mutation of a specific cysteine in D5 of gp130 C458A leads to constitutive heterodimerization with the  **LIFR**  and increased sensitivity towards  **LIF**  stimulation.  Ref: 12047380 Eur J Biochem, 2002
- These results show that even though human prolactinomas express gp130 and  **LIFR** , and respond to exogenous  **LIF** , albeit less than normal lactotrophs, they are largely devoid of  **LIF** .  Ref: 12574225 J Clin Endocrinol Metab, 2003
- The proliferative results confirmed that CNTF required homodimerization of the gp130, CNTF R and  **LIF**  R  [ **LIFR** ]  receptor subunit whereas Hyper CNTF required heterodimerization of the gp130 and  **LIF**  R  [ **LIFR** ]  receptor subunit.  Ref: 12765290 J Zhejiang Univ Sci< MedlineTA>J Zhejiang Univ Sci, 2003
- To elucidate the function of the two cytokine binding modules CBM of the leukemia inhibitory factor  [ **LIF** ]  receptor  **LIFR** , receptor chimeras of  **LIFR**  and the interleukin 6 receptor IL 6R were constructed.  Ref: 11812136 J Mol Biol, 2002
- Although all of 19 prolactinomas tested expressed gp130 and  **LIFR**  subunit mRNA and immunoreactive protein, only 3 of 19 prolactinomas expressed  **LIF**  mRNA.  Ref: 12574225 J Clin Endocrinol Metab, 2003
- Here we report that the expression of leukemia inhibitory factor  [ **LIF** ]  receptor  **LIFR** , one of the signaling molecules shared by several neuropoietic cytokines of the IL 6 family, is specifically upregulated in PC12 cells following treatment with NGF.  Ref: 12871977 J Biol ChemJ Biol Chem, 2003
- Either the NH 2 terminal chimera RILLIFdeltaI or the COOH terminal  **LIFR**  CBM chimera RILLIFdeltaII were replaced by the structurally related CBM of the IL 6R which does not bind  **LIF** .  Ref: 11812136 J Mol Biol, 2002
- The functional receptor complex of ciliary neurotrophic factor CNTF, a member of the gp130 family of cytokines, is composed of CNTF, the CNTF receptor alpha CNTFR, gp130 and the leukemia inhibitory factor  [ **LIF** ]  receptor  **LIFR** .  Ref: 12707266 J Biol Chem< MedlineTA>J Biol Chem, 2002
- Continuous treatment with Dex 10 8 mol L inhibited the endogenous production of IL 6,  **LIF** , OSM, IL 11R, and OSMR, but had no detectable effect on the expression of IL 11, CT 1, CNTF, CNTFR,  **LIFR** , or gp130.  Ref: 12110437 Bone, 2002
- The main function of the COOH terminal CBM of the  **LIFR**  is to position the NH 2 terminal CBM and the Ig like domain, so that these can bind to  **LIF** .  Ref: 11812136 J Mol Biol, 2002
- Both surface and glandular epithelial cells expressed maximal levels of  **LIF**  and  **LIFR**  protein, in biopsy samples showed fully developed pinopodes.  Ref: 12620495 Fertil Steril, 2003
- Here we studied the expression of cytokines IL 6,  **LIF** , IL 1beta and TNFalpha and cytokine receptors IL 6R,  **LIFR**  and Gp130 in the rat brain after kainic acid induced status epilepticus using Northern blot analysis and in situ hybridization histochemistry.  Ref: 12591161 Brain Res Mol Brain Res, 2003
- The leukemia inhibitory factor  [ **LIF** ]   **LIF** , cardiotrophin 1 CT 1 and oncostatin M OSM are four helix bundle cytokines acting through a common heterodimeric receptor composed of gp130 and  **LIF**  receptor  **LIFR** .  Ref: 12707269 J Biol Chem< MedlineTA>J Biol Chem, 2003
- Mapping and microsatellite marker development for the porcine leukemia inhibitory factor  [ **LIF** ]  receptor  **LIFR**  and epidermal growth factor receptor EGFR genes.  Ref: 12698007 Cytogenet Genome Res, 2002
- Before and after the appearance of pinopodes,  **LIF**  and  **LIFR**  immunostaining was less intense or faint.  Ref: 12620495 Fertil Steril, 2003
- **LIF**  was up regulated in the hippocampus, cortex and meninges after seizures, and  **LIFR**  mRNA was induced in the hippocampus and cortex.  Ref: 12591161 Brain Res Mol Brain Res, 2003
- The heterodimeric cytokine composed of the soluble ciliary neurotrophic factor receptor sCNTFR and the IL 6 family member cardiotrophin like cytokine CLC was recently identified as a new ligand for gp130 leukemia inhibitory factor  [ **LIF** ]  receptor  **LIFR**  complex Plun Favreau, H.  Ref: 11952795 Eur J Biochem, 2002
- OBJECTIVE To determine cell type specific expression of leukemia inhibitory factor  [ **LIF** ]   **LIF**  and  **LIF**  receptor  **LIFR**  proteins relative to formation of pinopodes in human endometrial samples.  Ref: 12620495 Fertil Steril, 2003
- Hyper CNTF efficiently induced dose dependent STAT3 phosphorylation and proliferation of BAF 3 cells stably transfected with gp130 and  **LIF**  R  [ **LIFR** ]  cDNAs.  Ref: 12071967 Eur J Biochem, 2002
- Because corticosterone is known to have a broad range of effects and  **LIF**  function has been associated with the hypothalamo pituitary adrenal axis, this study was designed to determine the role for  **LIFR**  in the fetus when exposed to the elevated maternal glucocorticoid levels of late gestation.  Ref: 12823859 Reprod Biol Endocrinol< MedlineTA>Reprod Biol Endocrinol, 2002
- Here, we report the construction and expression of a CNTF soluble CNTF receptor sCNTF R fusion protein Hyper CNTF with enhanced biological activity on cells expressing gp130 and leukemia inhibitory factor  [ **LIF** ]  receptor  **LIF**  R  [ **LIFR** ] , but not membrane bound CNTF R.  Ref: 12071967 Eur J Biochem, 2002
- 2 prolonged treatment with atypical antipsychotics may increase the anti inflammatory capacity of the serum in schizophrenic patients by increasing serum  **LIF**  R  [ **LIFR** ]  concentrations.  Ref: 11950553 Schizophr Res, 2002
- BACKGROUND Ablation of the low affinity receptor subunit for leukemia inhibitory factor  [ **LIF** ]   **LIFR**  causes multi systemic defects in the late gestation fetus.  Ref: 12823859 Reprod Biol Endocrinol< MedlineTA>Reprod Biol Endocrinol, 2002
- In contrast to other hematopoietic cytokine receptors, the leukemia inhibitory factor  [ **LIF** ]  receptor  **LIFR**  possesses two cytokine binding modules CBMs.  Ref: 12731865 Biochemistry, 2003
- BACKGROUNDLeukemia inhibitor factor  **LIF** , a member of the interleukin 6 cytokine family, has recently been shown to inhibit HIV 1 replication both in vivo and in vitro.OBJECTIVELIF and its corresponding receptors gp130 and  **LIF**  receptor alpha  **LIFR**  alpha were studied in lymphoid tissue LT to reveal potential systemic immunoregulatory effects during the course of HIV 1 infection.METHODSLIF, gp130,  **LIFR**  alpha and HIV 1 replicating cells were identified at the single cell level by immunohistochemistry and quantified by computerized in situ imaging in tonsil and lymph nodes biopsies LT from patients with primary HIV 1 infection PHI, chronic HIV 1 infection cHI, long term non progressors LTNP and HIV 1 seronegative controls.RESULTSLIF and its receptors, gp130 and  **LIFR**  alpha were significantly P &lt.  Ref: 12799551 AIDS< MedlineTA>AIDS, 2003
- Finally, treatment with exogenously added  **LIF**  stimulated IL 6,  **LIF** ,  **LIFR** , and OSMR, but had no other detectable effects.  Ref: 12110437 Bone, 2002
- The aim of this study was to assess the expression and regulation of  **LIF**  receptor  **LIFR**  and gp130 mRNA in mouse uterus during early pregnancy using in situ hybridization and reverse transcription polymerase chain reaction RT PCR.  Ref: 12203823 Mol Reprod Dev, 2002
